# Rare and Hungry: Feeding Ecology of the Golden Alpine Salamander, an Endangered Amphibian in the Alps

**DOI:** 10.3390/ani13132135

**Published:** 2023-06-28

**Authors:** Emma Centomo, Luca Roner, Marco Salvatori, Paolo Pedrini, Antonio Romano

**Affiliations:** 1 Ambito Biologia della Conservazione, Ufficio Ricerca e Collezioni, MUSE—Museo delle Scienze, Corso del Lavoro e della Scienza 3, I-38122 Trento, Italy; centomo.emma@gmail.com (E.C.); luca.roner@muse.it (L.R.); marco.salvatori@muse.it (M.S.); paolo.pedrini@muse.it (P.P.); 2 Dipartimento di Biologia, Università di Firenze, Via Madonna del Piano 6, I-50019 Firenze, Italy; 3 Consiglio Nazionale delle Ricerche—Istituto per la BioEconomia, Via dei Taurini 19, I-00100 Roma, Italy

**Keywords:** diet, herpetology, individual specialization, prey selectivity, optimal diet theory, distinct preference model, predator-prey system, *Salamandra atra aurorae*, trophic strategy

## Abstract

**Simple Summary:**

We investigated the trophic ecology of the Golden Alpine salamander (*Salamandra atra aurorae*), a rare and endemic amphibian found in a mixed temperate forest in northern Italy. We aimed to determine the salamander’s trophic niche, prey selectivity, and individual specialization in foraging. We analysed stomach contents from 53 salamanders obtained by stomach flushing technique and assessed prey availability through pitfall traps. The results revealed that the Golden Alpine salamander adopts a generalist feeding strategy at the population level but selectively prefers certain prey categories, such as Myriapoda and Hymenoptera (except Formicidae). Factors like prey size, movement ability, and degree of chitinization seem to influence food preference. The study also found significant inter-individual variation in dietary preferences, which was discussed in relation to optimal diet theory. Our research provides valuable insights into the diet of the Alpine salamander complex, suggesting similar feeding strategies between the subspecies.

**Abstract:**

Amphibians are considered critical species in the nutrient flow within and across ecosystems, and knowledge on their trophic ecology and niches is crucial for their conservation. For the first time we studied the trophic ecology of the rare and endemic *Salamandra atra aurorae* in a mixed temperate forest in northern Italy. We aimed to define the realized trophic niche, investigate the prey selectivity and explore possible levels of individual specialization. In summer 2022 we obtained stomach contents from 53 salamanders by stomach flushing and prey availability using pitfall traps. We used the Costello graphical method to analyse the realized trophic niche, and the relativized electivity index to study prey selectivity. Our results show that the Golden Alpine salamander adopts a generalist feeding strategy with positive selection of few prey categories (e.g., Myriapoda, Hymenoptera except Formicidae). Food preference seems to be driven by size, movement ability and chitinization of the prey. A high degree of inter-individual diet variation, modularity and clustering was found, describing a scenario that can be framed in a Distinct Preference model framework. This study gives new insights on the trophic ecology of the Alpine salamander complex, whose subspecies appear to adopt similar feeding strategies.

## 1. Introduction

Amphibians are one of the most threatened groups of vertebrates at the global level, with over 40% of known species listed as endangered, vulnerable, or critically endangered [1], and habitat loss, fragmentation, degradation, and alien species recognised as the main pressures [2,3]. As amphibians play a critical role in ecosystem functioning, their decline is expected to have severe consequences for ecological processes and even human welfare. By preying on a diverse array of invertebrates, being subject to predation, and utilising different environments across their life cycle, they play a pivotal role in nutrient flow within and across ecosystems. Knowledge on the feeding ecology of amphibians is therefore essential to inform conservation strategies and management plans to safeguard their survival and the ecological functions they provide. Dietary studies on amphibians have been crucial in elucidating their trophic interactions with other organisms in their respective ecosystems. Salamanders are one of the most diverse groups of amphibians, their feeding ecology varies greatly among species, habitats, and life stages [4], and they play important ecological roles in many terrestrial and aquatic ecosystems [5]. In certain environmental contexts, they have been shown to consume annually up to 5.8 kcal/m^2^ per salamander, with a significant impact on soil fauna [6]. Despite their ecological importance, the feeding ecology of many salamander species remains poorly understood.

*Salamandra atra,* commonly known as the Alpine salamander, is a small terrestrial and viviparous amphibian species that inhabits the Central-Eastern Alps and the Dinaric Alps, where some isolated populations occur [7]. This species is of particular interest to researchers due to its unique biology and ecological adaptations [[8,9], see also par. 2.1]. Four subspecies are generally recognised: the nominal *S. a. atra* Laurenti 1768, *S. a. prenjensis* Mikšić 1969, and the Italian endemics *S. a. aurorae* Trevisan 1982, and *S. a. pasubiensis* Bonato and Steinfartz 2005. Some studies have investigated the feeding ecology of *S. a. atra* and of *S. a. prenjensis* [10,11,12], but there is no information on the diet of *S. a. aurorae* and *S. a. pasubiensis*. *Salamandra atra aurorae*, the Golden Alpine salamander, due to its restricted geographic distribution (area of occupancy smaller than 20 km^2^) and its declining population trend [13], is assessed as “Endangered” (EN) in the IUCN Red List [14], and is included as a “priority taxon” in the European Union Directive 92/43/EEC (“Habitats Directive”). Storm VAIA, which caused millions of trees to fall in North-Eastern Italy in October 2018, represented a direct threat to the conservation of the Golden Alpine salamander both due to the strong impact on its range [13], and to the urgent post-event forest management. With the increasing use of heavy equipment, forestry practices, which compacts the soil and eliminates cavities and potential refuges, are considered as the main threat to this taxon [14].

We investigated the diet and feeding ecology of this subspecies of high biogeographic and conservation value. Understanding the feeding ecology of *S. a. aurorae* is important for several reasons. First, it can provide insights into the salamander’s role in the food web and its interactions with other species in the ecosystem. Second, knowledge of the diet and feeding behaviour can inform conservation efforts by identifying potential threats to its survival. Finally, a better understanding of the feeding ecology of *S. atra aurorae* can contribute to broader knowledge on the ecology of salamanders. In particular, our aims were threefold: (i) define the realized trophic niche and trophic strategy of this salamander at the population level (e.g., generalist or specialist); (ii) describe prey selectivity considering the environmental food availability; (iii) investigate possible levels of individual specialization in foraging. Specifically, we expected that the Golden Alpine salamander would show a generalist diet, given knowledge on the other subspecies (Prediction P1). However, we expected that it would show avoidance of certain unpalatable taxa and selection of nutrient-rich ones, as well as avoidance of small, fast, and flying prey, and selection of slower and bigger prey (Prediction P2). Considering previous studies about the trophic ecology of the Alpine salamander, we expected a high degree of inter-individual diet variation with major differences among the diets of individuals (Prediction P3).

## 2. Materials and Methods

### 2.1. Study Taxon

*Salamandra atra aurorae*, the Golden Alpine salamander, is an endemic subspecies of the Alpine salamander (*Salamandra atra*), isolated in the south-eastern Prealps, probably during the late Pleistocene [15]. The distribution of this salamander is limited to a restricted montane area located on the Sette Comuni plateau in the province of Vicenza (Veneto region) and Trento (Trentino Alto Adige region) [16,17].

Mixed forests dominated by silver fir (*Abies alba*) and beech (*Fagus sylvatica*), and to a lesser extent Norway spruce (*Picea abies*), represent the optimal habitat for this subspecies [9,17]. Structural features of the soil surface, distance from forest edges, and the availability of shelters are other important ecological requirements that influence its presence [9,17]. Like other Alpine salamanders, *S. a. aurorae* is a fully terrestrial and viviparous taxon: females, after a 2–3 year gestation period, give birth to just one or two fully developed young [18]. The species is most active in the warmest months (May–September) and is strictly dependent on meteorological conditions, with peaks of epigean activity during heavy rains [17,19,20].

### 2.2. Study Area

The study area is located on the Vezzena plateau (Trentino Alto Adige region), at about 1450 m a.s.l., (municipality of Levico Terme; 45°57′10″ N, 11°22′25″ E). The temperate humid climate of Trentino can be framed into the general climatic context of the other Alpine regions: the Lavarone-Vezzena area does not deviate significantly from this climatic panorama. However, the contiguity with the Venetian pre-Alps determines a high rainfall, with quite humid conditions compared to the average of Trentino, without significant dry periods during the summer [21]. At the macro-scale, alpine pastures, silver fir, beech, and plantations of spruce dominate the forest landscape, while at the fine-scale, the study area consists of a forest dominated by *Abies alba*, *Fagus sylvatica*, and *Picea abies*.

We selected a forest stand of 7.8 ha within which we drew 29 20 × 20 plots, covering a total area of about 1.16 ha; 9 of these plots were used in a previous study [9]. The plots have a considerably larger surface area than the home range of *S. a. aurorae* (mean 8 m^2^; [22]) and were separated by 30–50 m.

### 2.3. Sampling Predators

Sampling of salamanders occurred at the end of July (26–28–29 July 2022). Salamanders were collected in conditions of high detection probability (during or after rain events) in three nearly consecutive days. Captured animals were transported to the laboratory, 5 km from the sampling site, and since there is a significant increase in digestion rate with increasing temperature [23], salamanders were stored in a refrigerator at 4 °C in order to slow down prey digestion [11,24,25]. Salamanders were photographed with a digital camera situated perpendicular to the dorsal surfaces of the animals. Photographs of the dorsal patterns, which are unique for each individual in this taxon [22], were used to avoid recapture during subsequent sampling sessions, and to measure total length (TOTL: distance from the tip of the snout to the tip of the tail) with ImageJ^®^ software. Sex was determined through secondary sexual characters: adult males have a swollen cloaca, with a rounded outline when seen from the side, while females have a flat cloaca [26], with a posterior part of the trunk obviously swollen in the case of pregnant females [27]. In the present study, we only considered adult individuals (i.e., total length greater than 90 mm, according to Klewen [27]).

Stomach contents were obtained by stomach flushing [28,29], performed always by the same person using a 5 mL syringe (2–3 injections per salamander) connected to a flexible plastic tube. The flushing solutions were preserved in 70% ethanol. All animals were returned to their site of capture within a maximum of 6 h from their capture and no mortality was observed.

### 2.4. Prey Availability (Potential Trophic Niche)

Prey availability within the sampling site was assessed using 20 pitfall traps, one of the most widely used instruments for sampling soil and litter invertebrates [30,31]. This technique may overestimate more mobile fauna [32], however, prey mobility increases the detection probability by amphibians ([33,34]). For these reasons, it is reasonable to assume that such bias would not be misleading in the assessment of prey availability. Pitfall traps (500 cm^3^) were placed at ground level and a net was put over the top of the containers to prevent salamanders being trapped [11]. A sloped plastic top was placed above each pitfall to prevent flooding of the traps by rainfall. The containers were partially filled with a solution that prevents escape and aids conservation of trapped invertebrates (250–300 cm^3^ water with an addition of 500 mg of benzoic/acetic acid) [35,36]. The traps placed in 20 random trapping points within the salamander sampling area were active for four days immediately after predator sampling, in weather conditions comparable to those of salamander samplings (i.e., rainy days). The random spatial arrangement provides good coverage of the study area and ensured statistical independence of each trap [36].

The invertebrates obtained from environmental sampling and from stomach contents were subjected to the same identification procedure, using a dissecting microscope and taxonomic keys. All invertebrates have generally been identified at the Order or Class level, both partially digested invertebrates obtained from stomach flushing and those captured in the traps. Given the objective of investigating functional and dimensional categories (P2 prediction), we have distinguished ecologically dissimilar life stages of the same taxon (e.g., winged adults and terrestrial larvae) and, using the same criterion, related taxa with marked ecological differences (e.g., flying Hymenoptera were distinguished from walking ones, e.g., ants) [11,37,38]. Adult carabids, for their markedly larger size, were distinguished from other Coleoptera adults, while larvae were grouped into a single category. Throughout the entire manuscript, we will use this categorization for identified invertebrates.

### 2.5. Data Analysis

#### 2.5.1. Potential and Realized Trophic Niche

Prey diversity in the environment and in the diet was estimated through Simpson’s index (1-D; [39]), which measures ‘evenness’ of the community from 0 to 1, and 95% confidence limits calculated by bootstrapping [40]. Sex differentiation in diet was analysed by analysis of similarity (ANOSIM, a non-parametric test of significant difference between two or more groups), based on Bray–Curtis distances [41] with sequential Bonferroni correction. Data analyses were performed with the statistical package PAST version 4.03 [42].

#### 2.5.2. Trophic Strategy and Selectivity

The realized trophic niche was analysed with a modification of Costello’s graphical representation [43,44]. This method plots prey categories in a graph using two different variables: the *X*-axis represents the frequency of occurrence [FO], defined as the proportion of predators feeding on prey *i*, while the *Y*-axis displays the prey-specific abundance [P_i_], defined as the relative abundance of prey item *i* calculated on the total items found only in those individuals that fed on this prey category. The position of prey categories along the vertical axis sets out the feeding strategy of the predator: specialized when prey taxa have high P_i_ values and are projected in the upper part of the plot; generalist when all prey taxa have low P_i_ values and are projected in the lower part of the plot. Furthermore, diagonals of the graph allow the determination of the prey’s importance, as rare or dominant, and the contributions of BPC (between-phenotype component, namely the variation of resource use among individuals) and WPC (within-phenotype component, namely the variation of resource use by each individual) to the niche breadth.

This graphical approach is widely used to study the realized trophic niche of terrestrial and aquatic amphibians (e.g., [11,38,45,46,47]) and other taxa [48,49]. Costello’s graphical representation does not provide any information on the consumption and selection of prey in relation to their availability in the environment. Conversely, Vanderploeg and Scavia’s Relativized Electivity Index (E*; [50]), which is strongly supported by comparative evaluations [51], gives insights into the use of different prey types in relation to their abundance in the environment:

$$E^{*}=\frac{W_{i}-\frac{1}{n}}{W_{i}+\frac{1}{n}}$$

where$\;W_{i}=\frac{\left(r_{i}/p_{i}\right)}{\sum_{i}^{}(r_{i}/p_{i})}$ r*_i_* is the relative abundance of prey *i* in the diet, *p_i_* is the relative abundance of prey *i* in the environment, and *n* is the number of prey types. This index ranges from − 1 (negative selection) to 1 (positive selection), while a value of zero suggests prey consumption according to its availability (random feeding). As this index is particularly sensitive to the categories of prey with low environmental abundance, E* was calculated only for prey categories with more than three individuals sampled in the environment [47]. The 5th percentile of the absolute value of E* was set as a threshold electivity value (*u*), above and below which E* was considered different from zero [52].

#### 2.5.3. Comparison with Other Studies

For food availability comparisons between Alpine salamander subspecies, we rearranged the potential availability and the consumed prey into comparable taxa (17 and 16, respectively in our study). Since the study of Šunje et al. [12] differed in the number of pitfalls, sampling period and number of sites, a comparison of the dominant taxa proportions is more appropriate, as they do not change when compared with data among sampling periods of different length [36].

#### 2.5.4. Inter-Individual Diet Variation

We used network analysis to assess the variation of diet among individuals. We created a network connecting individual salamanders with their prey items, and each link represented the strength of the relationship, namely the frequency of use. We calculated the centrality of the network nodes, and therefore, the importance covered by each node in the web, through the Davidson-Harel simulated annealing algorithm [53]. We then calculated four network metrics to quantify the features of this predator-prey system: degree of diet variation, nestedness, modularity (with corresponding number of modules), and degree of clustering. The degree of inter-individual diet variation was measured through the index *E* [54]: based on the pairwise overlap of individual diets and its average in the sampled population, the index *E* can go from 0 when all sampled individuals have identical diets, to 1 when diet variation is at its maximum. Nestedness in the trophic ecology of a species occurs when the diets of some specialist individuals include only a subset of the prey consumed by the more generalist individuals. We measured nestedness with NODF, a metric based on overlap and decreasing fill [55], that can take a value from 0 to 100 (minimum and maximum nestedness). Modularity *Q* can be recorded when it is possible to separate a population into groups of individuals with similar diets, and we calculated it with the Beckett’s algorithm [56]. Since this algorithm is stochastic, we repeated it 1000 times and retained the network partitioning that maximised modularity [56]. The index Q can range from 0, indicating that no link exists between nodes in the same module, to 1, when all network links are interactions within modules. The degree of clustering *Cws* developed by Araújo et al. [54] measures the degree to which predators tend to be grouped into distinct clusters according to their diet niche overlap, and though mathematically different from modularity, it is conceptually very similar. Degree of diet variation, nestedness, and modularity/clustering can also arise for stochastic processes or sampling bias; we therefore compared the observed values of these metrics against 10,000 bootstrapped simulations with null models. The calculations were made in R through the packages Bipartite and RinSp.

## 3. Results

### 3.1. Potential and Realized Trophic Niche

**Figure 1:**
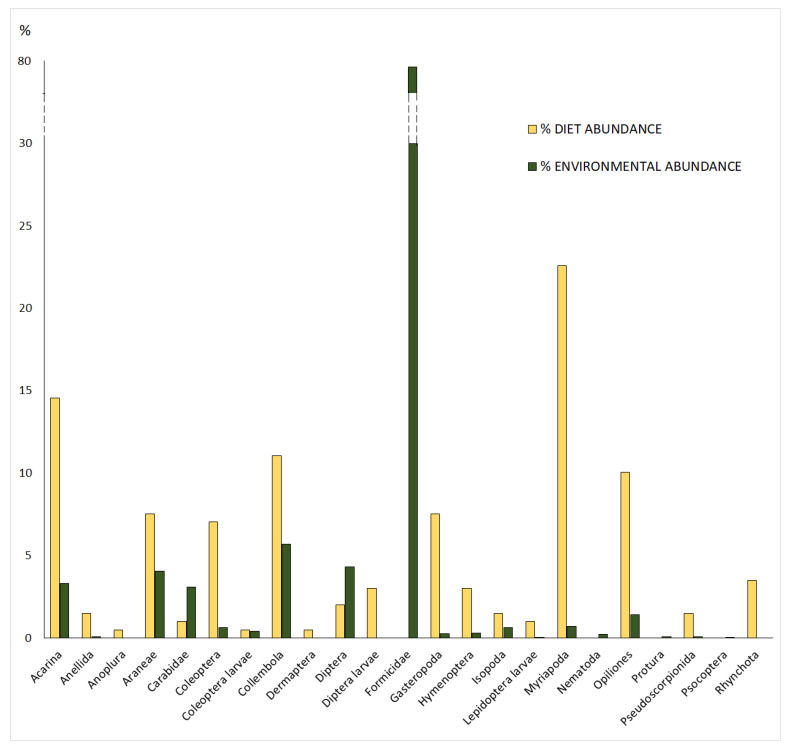
Comparison between the percentage abundance of prey categories in diet and in the environment.

In 3 sampling days, 53 adult salamanders (34 males and 19 females, of which 11 were pregnant) were captured and stomach flushed with 44 positive results, six individuals with no prey in their stomach and three individuals with only indeterminate prey items (prey that could not be identified because of the high digestion level). The 199 analysable prey items found in the stomach were divided into 19 categories belonging to 16 taxa (*n* = 41 indeterminate; Figure 1), with an average of 3.98 ± 4.35 prey/stomach (mean ± s.d.; *n* = 50; range = 0–24). There was no difference in the diet composition between the sexes (ANOSIM, *n* = 44; global R = −0.07, *p* = 0.95) and between pregnant and non-pregnant females (ANOSIM, *n* = 15; global R = −0.01, *p* = 0.49). Environmental sampling produced a total number of 2867 invertebrates representing 20 prey categories belonging to 17 taxa (Figure 1).

The diversity of prey categories in the environment and diet calculated as Simpson’s index is shown in Table 1.

**Figure 2:**
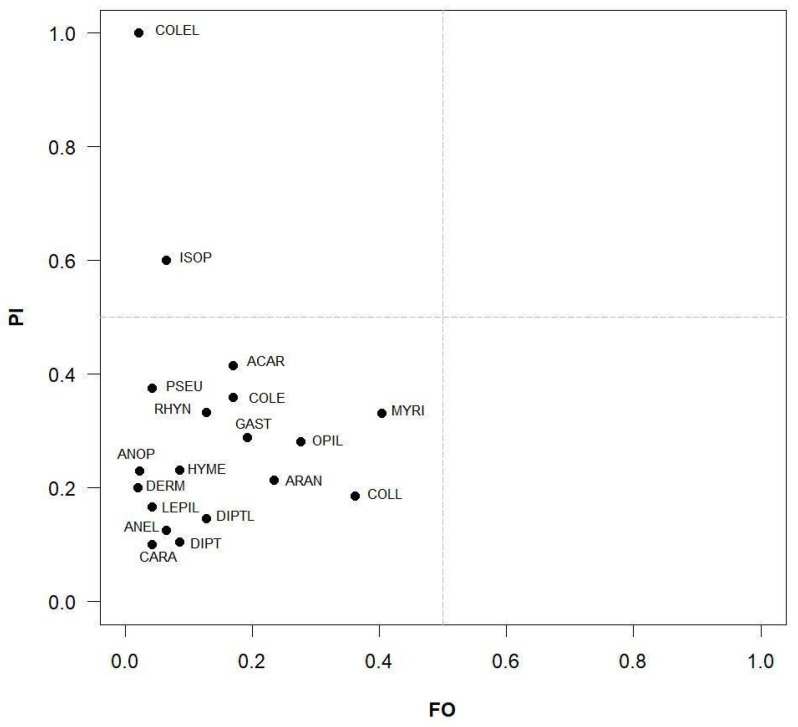
Costello’s modified plot [43,44] describing the trophic strategy of *Salamandra atra aurorae*. Legend: ACAR, Acarina; ANEL, Anellida; ANOP, Anoplura; ARAN, Araneae; CARA, Carabidae; COLE, Coleoptera; COLEL, Coleoptera larvae; COLL, Collembola; DERM, Dermaptera; DIPT, Diptera; DIPTL, Diptera larvae; GAST, Gastropoda; HYME, Hymenoptera (not Formicidae); ISOP, Isopoda; LEPIL, Lepidoptera larvae; MYRI, Myriapoda; OPIL, Opiliones; PSEU, Pseudoscorpionida; RHYN, Rhynchota.

The analysis of the Costello’s modified plot (Figure 2) clearly shows that this *Salamandra atra aurorae* population behaved as a generalist predator, confirming our prediction P1. In fact, the majority of prey categories are located in the lower left part of the graph (FO and PI < 0.5), with only two (Coleoptera larvae: PI = 1, FO = 0.02; Isopoda: PI = 0.6, FO = 0.06) in the upper left quadrant (PI > 0.5; FO < 0.5).

### 3.2. Trophic Selectivity

**Figure 3:**
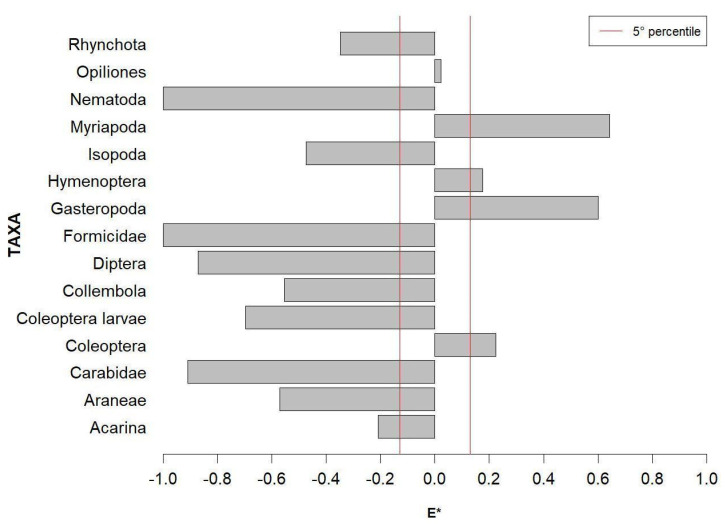
Relativized Electivity index E* based on trophic availability. Within the red vertical lines, values are not statistically significant.

The analysis of the relativized electivity index E* showed that salamanders positively selected only Myriapoda, Hymenoptera, Gastropoda, and Coleoptera, while all other prey categories were apparently avoided (Figure 3). Opiliones was the only prey consumed in proportion to environmental availability, with an E* value within the significance threshold.

Salamanders operated a negative selection on two of the commonest available prey items (Collembola and Formicidae), while, among the most abundant preyed taxa, a positive selection was only observed on Myriapoda and Gastropoda.

### 3.3. Comparison with Other Studies

The diversity of prey taxa in the environment of *S. a. aurorae* (17 taxa) was numerically very similar to that found both in the habitat of *S. a. atra* (15; [11]) and higher or similar to *S. a. prenjensis* (from nine to 16 taxa across four populations; [12]). The dominant taxa proportion, Coleoptera (22.3%), Diptera (15.8%), and Hymenoptera (15.7%) were the most represented taxa in the study by Šunje et al. [12], while in our study, Hymenoptera (Formicidae), Collembola, and Diptera were dominant. On the other hand, the total amount of prey sampled in the environment varied markedly. For the Golden Alpine salamander, it was almost five times greater than that detected for the nominal subspecies, which was studied in high mountain environments [11], and almost double that detected for *S. a. prenjensis* based on sampling from four different areas [12]. The average number of ingested prey by Golden Alpine salamander (3.98 ± 4.35, N = 50) is greater than that reported for *S. a. atra*, both in comparison with the north-eastern Italian population (2.78 ± 0.75, N = 45; [11]), and in the two populations of the Austrian Alps (2.59 ± 1.85, N = 39; [10]). The mean value is also greater than that reported for the four populations of *S. a. prenjensis* (3.16 ± 0.55, N = 264; [12]). However, the Simpson index (1-D) revealed a diversity of prey in the environment lower than that calculated for *S. a. atra* (0.44 vs. 0.80 respectively; [11]), and to that recalculated for *S. a. prenjensis* on standardized potential prey, since the index is sensitive to the number of taxa (*i*) used (0.44 vs. 0.87, all populations of Šunje et al. [12] pooled respectively; the Simpson index among populations varied from 0.80 at Prokletije, to 0.85 Čvrsnica). Regarding the preyed invertebrates, the number of prey taxa eaten by *S. atra aurorae* (16) is higher than that found for *S. atra atra* (10; [11]), but it is similar to that found for *S.a. prenjensis* (from 14 to 18 taxa for four populations; [12]). Indeed, by applying to *S. a. prenjensis* the same categorization of prey that we applied, we verified that the diversity of preyed taxa is very close to what we estimated for *S. a. aurorae* (Simpson Index, 1-D = 0.88 vs. 0.90 for *S. a. aurorae* and for all pooled populations of *S. a. prenjensis* respectively, [12]; the index among populations varied from 0.85 for the site Prokletije to 0.90 for the site Gorski Kotar).

### 3.4. Inter-Individual Diet Variation

**Figure 4:**
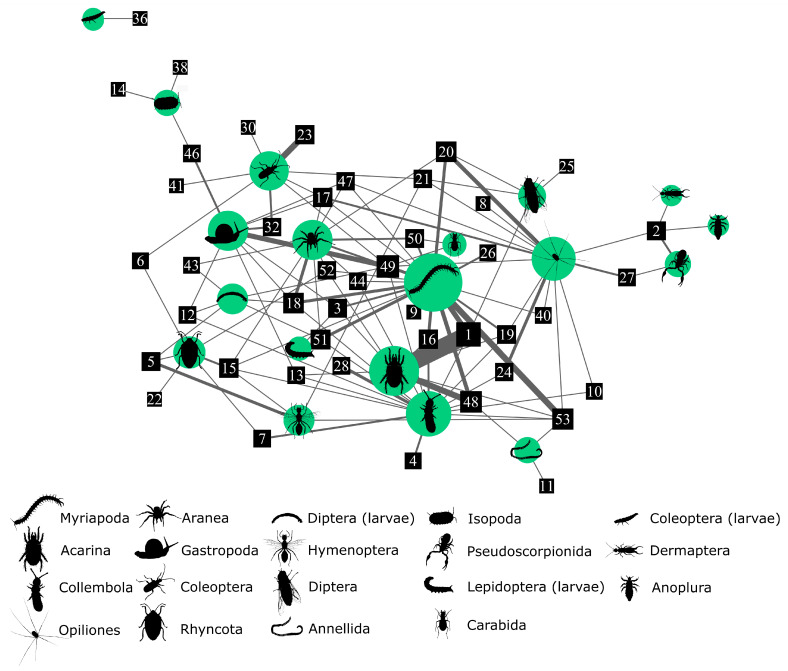
Network of predator-prey interactions based on diet analysis of the Golden Alpine salamander. Individual salamanders are represented by black squares and an identity number, while prey taxa are shown by their silhouettes within green circles. The centrality of each node reflects the general number and strength of connections with all the other nodes in the network, while its size indicates the number and weight of its direct connections. The width of the edges linking the nodes represents the number of predator-prey interactions.

Myriapoda, Aranea, Diptera larvae, and Lepidoptera larvae were the prey items that occupied the central nodes of the network, whereas Coleoptera larvae, Isopoda, Dermaptera, and Anoplura were the most peripheral ones (Figure 4).

**Figure 5:**
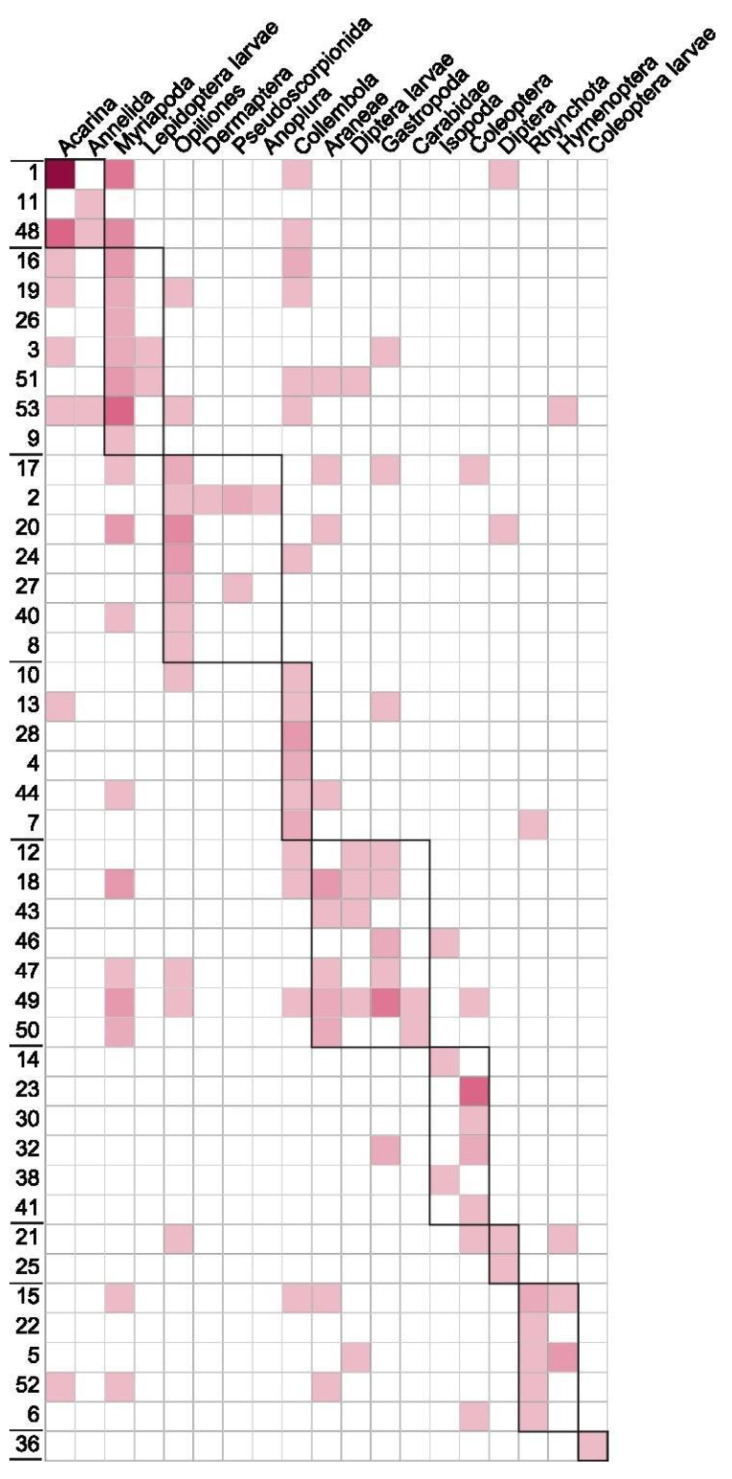
Weighted matrix of predator-prey interactions. Predators (salamanders) are represented as rows and prey taxa as columns, and the number of interactions is indicated by the color gradient (light pink: few interactions, dark red: many interactions). Cells have been subdivided into modules, enclosed by black rectangles, through the Beckett’s algorithm. Individual salamanders within the same module present more similar diets than between different modules.

The degree of diet variation indicated a strong variability among the diets of sampled individual salamanders (*E* = 0.86, *p* < 0.001) that were also subdivided into different trophic groups (nine modules detected, Figure 5). The network was indeed characterized by positive modularity (*Q =* 0.47, *p* < 0.001) and degree of clustering (*Cws =* 0.29, *p* < 0.001). Degree of diet variation *E*, modularity *Q*, and degree of clustering *Cws* were all markedly higher than expected under the 10,000 bootstrap simulations with null models, whereas nestedness was remarkably lower than expected under the null hypothesis (*NODF* = 6.45, *p* < 0.001; see also Figure S1).

## 4. Discussion

Many populations of *Salamandra atra* are known for living in continuous and large forests; however, among the four recognized subspecies, the Golden Alpine salamander is the only one restricted to forest environments, mainly mixed coniferous and deciduous forests [57,58]. The altitudinal range is narrower in comparison with the nominal subspecies (1200–1850 m asl). In the animal world, differences in dietary ecology may be exhibited among subspecies (e.g., [59,60]) or populations (e.g., [61]). Considering that the Golden Alpine salamander occurs in a different ecological context in comparison to the other Alpine salamander subspecies, its diet could reflect this ecological uniqueness in terms of diversity and abundance of consumed prey.

### 4.1. Prey Availability and Realized Trophic Niche

We did not find significant differences in trophism between the two sexes, as found in the other subspecies of this species [11,12], and in other salamander species (e.g., [38,46,47]). In *Salamandra atra*, pregnant and non-pregnant females did not show differences in feeding (this study; [11]), probably because they have a prolonged viviparous gestation period of 2–3 years [19], which is too long to avoid feeding. Costello’s graphical method (Figure 2) identified a generalized trophic strategy for the Golden Alpine salamander, confirming our prediction P1. However, the position of Coleopteran larvae and Isopoda in the upper-left part of the graph (i.e., the most abundant prey category in the diet) indicates they were consumed in high abundances only by a few individuals, suggesting a limited within-phenotype component with a certain degree of individual specialization. A similar pattern was found in *S. a. atra*, where Diptera larvae occupied the same position of the coleopterans [11]. The superclass Myriapoda represents an important trophic resource for many individuals, both for the nominal subspecies [11] and for the Golden Alpine salamander (this study).

Compared to other studies on Alpine salamanders, the lower Simpson Index value of prey availability is due to the high number of ants present in our traps, which greatly influences the equipartition of individuals among taxa and, therefore, the calculation of the index. Conversely, the higher number of prey ingested by Golden Alpine salamander in comparison to four populations of *S.a. prenjensis* could be due to the procedure followed by Šunje et al. [12] (described in [62]) that applied only one flushing per salamander, potentially influencing the data collection. The diet of the *S. a. aurorae* appears varied and abundant compared to the other Alpine salamanders, but not equivalent proportionally to the availability found in its natural environment. Consequently, *S. a. aurorae*, along with the other subspecies, may be considered a frugal consumer, unlike other salamander species (e.g., [47]). As suggested for the nominal subspecies [11], the limited number of food items in the salamander stomachs cannot be merely attributed to the scarce food availability, but probably also to the dimensional selectivity related to morphological characteristics of the predators and their predation strategy (see Section 4.2). The avoidance of some small-sized taxa (e.g., Collembola) by Golden Alpine salamanders may indicate a preference for dimensional selectivity. This strategy may be more efficient, as consuming fewer but larger prey items can provide a better energy gain per time unit compared to consuming many smaller prey. Ultimately, this approach allows the predator to maximise the energy intake and prioritise the most valuable food sources [63,64].

### 4.2. Trophic Selectivity

Many studies that have analysed the diet of salamanders have not taken account of the prey availability in the environment. Consequently, the salamander’s ability to selectively feed on certain prey items remains poorly understood. However, the trophic selectivity of salamanders has been increasingly studied in recent years [11,12,25,38,47,65,66,67]. Our analysis clearly shows that the Golden Alpine salamander does not prey in proportion to environmental availability. The salamander carries out a significant positive selection towards Myriapoda and Gastropoda, and, less markedly, towards flying Hymenoptera and Coleoptera (Figure 3). Myriapoda and Gastropoda seem to play a key role in the diet of the Alpine salamander, since the nominal subspecies, in a markedly different environment, shows the same preferences, significantly selecting these taxa [11]. Even the subspecies *S. a. prenjensis*, sampled in four different environments, ingests Myriapoda and Gastropoda more frequently than expected from their presence in the potential trophic niche [12]. Furthermore, the Myriapoda are significantly associated with the population sampled in the Gorski Kotar site, the closest to our site in terms of habitat, with a positive selection on Chilopoda [12]. The general trend is a positive or random selection towards relatively slow and/or large prey items, and a negative selection towards smaller, faster and/or flying prey, in agreement with the realized trophic niche, and partially confirming our prediction P2. The degree of body chitinization seems to be a secondary criterion in the predatory choice for the Golden Alpine salamander, as some of the positively selected prey have a high degree of chitinization and are only partially or slowly digested by the salamander, as emerged also by the observation of the stomach contents. The higher cost related to digestion is probably compensated by the energy saving in the search and capture phase, and by the energy intake given by the large size of these taxa (see also Section 4.1).

The distinction between the functional categories of larvae and adults has been useful to highlight how the same taxon can represent a very different potential prey during its life cycle for predators such as salamanders (e.g., [11,38,68]). The Golden Alpine salamander indeed positively selects only the adult Coleoptera, while avoiding their larvae. In this case, however, the result is unexpected: the larvae, generally slow and with non-chitinized bodies, should be favoured according to the identified predation strategy. The difficulty of pitfalls to sample poorly active prey [36] and the underestimation of prey composed of soft tissues, rapidly digested and difficult to detect with the stomach flushing technique [34], could partly explain this result. A plethora of other invertebrate taxa were not found in stomachs or negatively selected. Excluding red ants, whose chemical defenses (e.g., formic acid) make them generally unpalatable [69], Collembola were the most abundant potential prey not commonly eaten. Terrestrial or semi-terrestrial salamanders may prey on springtails or may have a diet specialized on them (e.g., *Salamandrina*; [38,47]). Capturing springtails is, however, challenging due to their possession of a furcula, which serves as an escape mechanism enabling them to rapidly jump and evade predators [70]. They are considered highly energetic prey because they possess a lower degree of chitinization [71]. Golden Alpine salamanders, unlike *Salamandrina,* do not extend the tongue to capture prey, making the capture of springtails particularly ineffective. Collembolans are the primary decomposers of plant matter in temperate forests, and they exist in high numbers in the soil [72]. Probably the ingestion of Collembola by the Golden Alpine salamanders may be the consequence of occasional consumption during foraging activity, as occurs with other small prey, including plant material such as pine or fir needles, which was frequently found in their stomach contents.

With regards to the remaining negatively selected prey, it is noteworthy that the consumption of prey by amphibians is heavily influenced by their morphological and physiological characteristics [33]. Additionally, there is a well-established positive correlation between the size of Urodela predators and their prey [73]. It is possible that the salamander’s relatively unsophisticated predation strategy, which may not be particularly effective in catching agile prey, also represents a limiting factor in its ability to consume small-sized prey. The relatively clumsy foraging style appears to be typical of Alpine salamanders (this study; [11,12]), suggesting that significant electivity indices could reflect predatory ability/inability, rather than actual preference or dislike. Abundance of most invertebrates depends on the presence of dead wood on the soil surface [74]. In particular, Myriapoda are more abundant close to wood debris [75], and they are also positively related to advanced states of wood decomposition [76]. Hymenoptera are related to dead wood as well, with parasitoid species more abundant near newly dead wood of fine diameter [77]. Preference and importance of specific prey’s taxa (Myriapoda and Hymenoptera) pointed out by our results represent useful information to proper forest management. These practices will have a double effect, considering that dead wood piles, in particular fine woody debris, have been shown to play a key role for Golden alpine salamander presence [9].

### 4.3. Inter-Individual Diet Variation

The collective trophic niche of a population is considered to be the outcome of adding up the trophic niche of each individual. However, the individual trophic niche can vary greatly. This means that even a population of generalist species may consist of individuals with varying levels of specialization, feeding on different types of prey and utilizing diverse trophic strategies [78,79]. Although *S. a. aurorae* exhibited a generalist diet at the population level, the high degree of diet variation indicates major differences among the diets of analysed individuals (confirming our prediction P3), as observed in other studies of the trophic ecology of Alpine and other salamanders [11,12,37,68,80]. Inter-individual diet variation can be explained in light of the optimal diet theory (ODT) [81,82] that accounts for three different models which can explain how individual specialization arises. Concerning our network, there are two possible suitable models: the Competitive Refuge (CR) model and the Distinct Preference (DP) model. In the CR model, individuals share the preferred prey and, under intraspecific competition, they expand their trophic niche to other less profitable prey, according to the individual preference. In the DP model, individuals are clustered in groups that exhibit different favoured food resources, according to different ethological and phenotypical group characteristics: in case of preferred resource decline, low availability or high intraspecific competition, foragers can expand their trophic niche and increase diet overlap, including alternative options (e.g., [83]). Modularity and nestedness are the most important variables that can elucidate the causes of inter-individual diet variation, according to the different scenarios described by the ODT theory. The first is observed when the diet of some individuals can be pooled in clearly defined subgroups, within which individual diets are very similar. The second is observed when some more specialized individuals consume a group of prey that is a subset of the diet of more generalist individuals. The diet of individual salamanders in our study is characterized by a high degree of modularity and clustering, while nestedness is low: this condition, uncommon in dietary studies [83], is also reported for *S. atra* population studied by Šunje et al. [12]. However, data obtained from a Dolomites population of the nominal subspecies indicate a moderate nestedness, whereas modularity is still high [11]. The high modularity degree in our network can be explained by both CR and DP models. Indeed, a significant modular network can emerge in the CR model when food resources are limited and intraspecific competition is intense, driving the foragers to include different prey types in realized trophic niches, causing the rise of different clusters. A high level of modularity can also emerge in a DP model framework: under the condition of abundant food resources and low intraspecific competition, foragers can specialize on individual preferred items. The low nestedness obtained from our analysis demonstrates a low overlap between individual trophic niches belonging to different modules. The CR model predicts a nested network when food availability is high and intraspecific competition is low, whereas in the DP model, nestedness arises when competition increases and food availability is low. Our results suggest little overlap between individual trophic niche and a consequent low intraspecific dietary competition, and the high prey availability we measured in the study area further corroborates this conclusion (see Section 4.1). Ultimately, the modularity and nestedness pattern observed in our framework, namely scarce trophic competition and high prey availability, are in agreement with the DP model postulated by the ODT theory. Indeed, the DP model predicts a modular network when competition is low and food resources abundant, with a shift toward a nested network when competition arises and food items are scarce. However, the inter-individual diet variation model we found derives from a limited sampling period with only one flushing per individual. Therefore, these limits raise a relevant question: is this the standard individual feeding habit of the Golden Alpine salamander, or does it represent merely a snapshot? To answer this question, we need to understand if relevant variables, like competition and trophic availability, can change significantly within and among the annual seasons, since changes in intraspecific competition and in food availability can lead to a shift in dietary pattern [82,84]. Given that population density of the Golden Alpine salamander cannot increase significantly due to the particularly low reproduction rate [18], as well as the low spatial overlap among individuals (mean home range = 8 m^2^, range 0–32; [22]), it is reasonable to assume a stable or very low increase of competition among foragers within and among seasons. Midsummer season represents one of the peaks of soil arthropod biomass and diversity, both in agricultural landscapes [85] and in Alpine forest habitats [86,87]. In particular, the period between June and July is characterized by high arthropod abundance in the subalpine forest context [86,87,88]. As shown by Costa et al. [89] resource diversity, rather than abundance, can enhance the emergence of inter-individual diet variation and modularity in salamanders’ prey-predator networks. The high inter-individual diet variation and modularity degree in our study could then change among different moments in the activity season. However, our sampling was performed in conditions of both soil arthropod diversity and salamanders’ activity peak during the season. For all these reasons, and considering data obtained by inter-individual diet variation (low diet overlap and high modularity in DP model framework), we are confident that we sampled salamanders and stomach contents in the most favourable dietary condition, leading thus to a description of the Golden Alpine salamander’s diet that reflects different individual ability or preference to different prey items. Furthermore, given that this is the third study that pointed out an inter-individual diet network in *S. atra* [11,12], the pattern observed seems widespread in different subspecies and habitats.

## 5. Conclusions

This is the first study on the diet of the Golden Alpine salamander, and it revealed that this urodele displays a generalist feeding strategy at the population level, but with a significant degree of inter-individual variation in dietary preferences that can be framed in a DP model framework. Salamanders showed a clear preference for certain prey categories (e.g., Myriapoda, Hymenoptera, Gastropoda, Coleoptera), while almost ignoring all other prey categories. Interestingly, the salamanders exhibited a negative selection for two of the most abundant prey categories, Collembola and Formicidae, despite their high trophic availability. Such knowledge is essential for understanding the ecological role of this salamander in its ecosystem and for developing conservation strategies. Further research could shed light on the underlying mechanisms driving the observed inter-individual variation in diet, and how it relates to the individual’s ecology and life history. The storm VAIA in 2018 severely impacted the forest habitat of the Golden Alpine salamander. Our ongoing research is trying to understand if the impact of the storm VAIA had consequences on the trophic availability and on the feeding strategy adopted by this endemic and rare salamander. Last but not least, diet preferences that emerged from our study can also provide relevant knowledge for appropriate forest management implementation. The guidelines are to make allowance for ecological requirements of Golden alpine salamanders’ prey, improving management that favours new and old dead wood piles accumulation.

## Figures and Tables

**Table 1:** Diversity index of the prey in environment and in the diet.

Simpson Diversity Index	1-D (95% C.I.)	Taxa
*Environmental availability*	0.44 (0.42–0.46)	17
*Stomach content*	0.88 (0.86–0.90)	16

## Data Availability

The data presented in this study are available from the corresponding author upon reasonable request.

## Supplementary Materials

The following supporting information can be downloaded at: https://www.mdpi.com/article/10.3390/ani13132135/s1, Figure S1: Results of the 10,000 bootstrap simulations with null models for nestedness (upper left), index of diet variation (upper right), modularity (lower left), and degree of clustering (lower right). Histograms show the distribution of the simulated values and the vertical red dotted lines indicate the observed value for each metric. Data derive from diet analysis of the Golden Alpine salamander.
