# Supplementary material for: Rare and Hungry: Feeding Ecology of the Golden Alpine Salamander, an Endangered Amphibian in the Alps
Source: Animals (Basel). 2023 Jun 28;13(13):2135. doi: 10.3390/ani13132135 (PMC10339940; doi:10.3390/ani13132135)
Supplement: Supplementary file 1 [file animals-13-02135-s001.zip › animals-2445965-supplementary.pdf]

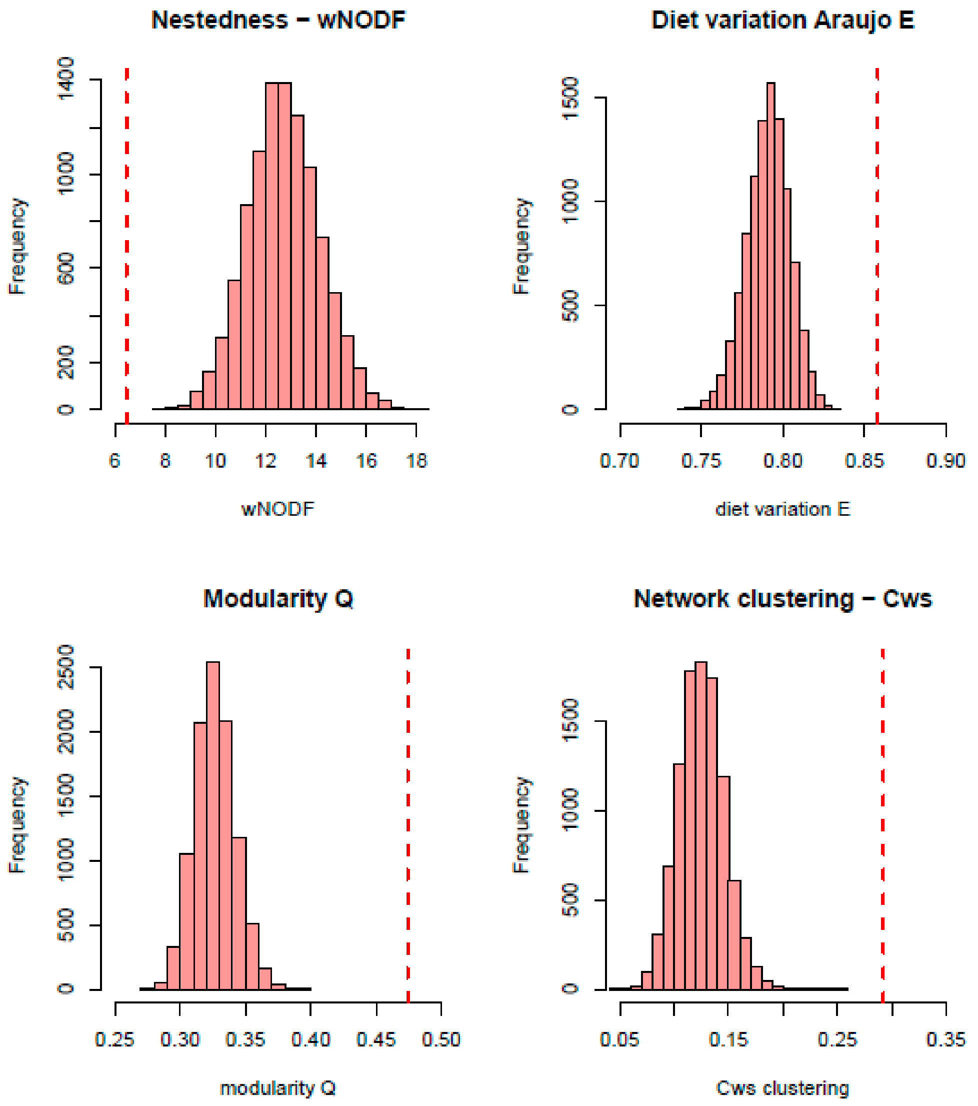

Figure S1. Results of the 10,000 bootstrap simulations with null models for nestedness (upper left), index of diet variation (upper right), modularity (lower left), and degree of clustering (lower right). Histograms show the distribution of the simulated values and the vertical red dotted lines indicate the observed value for each metric. Data derive from diet analysis of the Golden Alpine salamander.
